# Effect of Reduced Centrifugation Time on Leukocyte, Platelet, and Growth Factor Levels in Platelet-Rich Fibrin (PRF) Prepared Using Low-Speed Relative Centrifugal Force (RCF): An Ex Vivo Study

**DOI:** 10.7759/cureus.100846

**Published:** 2026-01-05

**Authors:** Shankar S Menon, Biju Balakrishnan, Arun Kurumathur Vasudevan, Maya Rajan Peter, Reshma Suresh

**Affiliations:** 1 Periodontics, Amrita School of Dentistry, Amrita Vishwa Vidyapeetham, Kochi, IND

**Keywords:** centrifugation time, leukocytes, periodontal regeneration, platelet-rich fibrin, platelets, tgf-β, vegf

## Abstract

Background: Platelet-rich fibrin (PRF), a second-generation autologous biomaterial, enhances periodontal regeneration through its fibrin matrix, which is enriched with leukocytes, platelets, and growth factors such as vascular endothelial growth factor (VEGF) and transforming growth factor-beta (TGF-β). Centrifugation parameters critically influence the regenerative composition of PRF, with low-speed relative centrifugal force (RCF) protocols improving both cellular and molecular yields. However, the impact of centrifugation time remains underexplored. This study investigates whether reducing centrifugation time from 8 to 4 minutes at 44 g (600 rpm) optimizes PRF’s regenerative profile for periodontal applications.

Methods: Peripheral blood from 10 healthy male volunteers (aged 20-30 years) reporting to a tertiary care center was processed into fluid PRF using two protocols: 8-minute and 4-minute centrifugation at 600 rpm. Leukocyte and platelet concentrations were quantified via flow cytometry, while VEGF and TGF-β levels were measured using enzyme-linked immunosorbent assay (ELISA) at 1 and 24 hours post-clotting. The Wilcoxon signed-rank test assessed differences between protocols (p ≤ 0.05).

Results: Reducing centrifugation time to 4 minutes significantly increased leukocyte (median: 0.04-0.07 ×10³ cells/µL, p = 0.008) and platelet (median: 578-691 ×10³ cells/µL, p = 0.005) concentrations. VEGF levels showed a near-significant rise (median: 4.19-6.10 pg/mL, p = 0.051), while TGF-β exhibited a non-significant upward trend (median: 35.57-36.43 ng/mL, p = 0.594). These enhancements suggest improved cellular retention and growth factor release.

Conclusion: A 4-minute centrifugation at 600 rpm suggests improved cellular retention and trends toward higher growth factor concentrations by enhancing leukocyte, platelet, and VEGF concentrations, with a modest TGF-β increase. This protocol offers potential biochemical enhancement with improved chair-side efficiency, warranting further clinical validation.

## Introduction

The evolution of regenerative dentistry has transformed periodontal therapy, shifting from basic wound management to advanced autologous biomaterials that harness the body’s innate healing mechanisms [1]. Platelet-rich fibrin (PRF), a second-generation platelet concentrate, has emerged as a cornerstone in periodontal regeneration because of its fibrin matrix enriched with platelets, leukocytes, and multiple growth factors [1,2]. Unlike platelet-rich plasma (PRP), PRF eliminates anticoagulants, relying on a single centrifugation step to form a natural clot rich in regenerative components [3]. Its sustained release of growth factors, such as transforming growth factor-beta (TGF-β) and vascular endothelial growth factor (VEGF), supports angiogenesis, matrix synthesis, and tissue remodeling, making it valuable for applications including socket preservation, management of gingival recession, and intrabony defect repair [4,5].

The regenerative efficacy of PRF depends largely on its preparation protocol, particularly the centrifugation parameters that determine its cellular and molecular composition [5,6]. Earlier protocols used high relative centrifugal force (RCF) values to maximize platelet and leukocyte entrapment, but over-compaction of fibrin and impaired growth factor diffusion prompted a move toward lower relative centrifugal forces [5,7]. The Low-Speed Centrifugation Concept (LSCC), introduced by Choukroun and Ghanaati, showed that reducing RCF enhances cellular yields and growth factor release, improving PRF’s biological performance [5].

However, centrifugation time remains underexplored as a variable for optimizing PRF preparation. Reducing centrifugation time offers distinct advantages for periodontal applications. A briefer preparation preserves cellular integrity while elevating growth factor availability, potentially accelerating angiogenesis and matrix synthesis, which are critical processes for periodontal tissue restoration [8-10]. This enhancement could improve clinical outcomes in defect regeneration and socket preservation, where enriched PRF matrices have demonstrated superior healing capacities [11,12]. Moreover, a truncated protocol increases procedural efficiency, enabling rapid chair-side preparation without compromising the clot’s structural robustness. By assessing platelet and leukocyte concentrations alongside TGF-β and VEGF levels, this investigation seeks to quantify these improvements, providing a comprehensive evaluation of PRF’s regenerative potential. This approach aligns with the broader pursuit of optimizing PRF, ensuring it meets the demands of periodontal therapy with heightened efficacy and practicality [5,13,14].

This study investigates the effect of reducing centrifugation time from 8 to 4 minutes at a moderate RCF of 44 g (600 rpm) on PRF’s composition, hypothesizing that a shorter duration enhances leukocyte, platelet, TGF-β, and VEGF concentrations. By preserving cellular integrity and amplifying growth factor availability, this adjustment could refine PRF’s regenerative potential for periodontal applications, while improving chair-side efficiency. The primary endpoints were leukocyte and platelet concentrations, while secondary endpoints were TGF-β and VEGF concentrations measured cumulatively over 24 hours. The investigation builds on LSCC principles, focusing on time optimization to balance clot formation with enhanced regenerative yields, addressing a critical gap in PRF protocol development [15-17].

## Materials and methods

The present study followed the ethical principles outlined in the Helsinki Declaration of 1975, as revised in 2013, and approval from the institutional ethics committee was obtained vide no. ECASM-AIMS-2024-308, dated 16-07-2024. This ex vivo experimental study evaluated the impact of reducing centrifugation time on fluid PRF composition at a constant low-speed RCF of 44 g (600 revolutions per minute (rpm)). RCF was calculated as g = 1.118 × 10⁻⁵ × radius (11 cm) × rpm², confirming 44 g at 600 rpm.

Two protocols were compared, Protocol I (8 minutes) and Protocol II (4 minutes), enabling direct assessment of the effect of time on leukocyte, platelet, TGF-β, and VEGF concentrations. The design followed LSCC principles, aligning with prior PRF optimization studies. The RCF of 44 g (600 rpm) was selected based on LSCC principles to optimize cellular yields [5], with 8 minutes as the standard protocol and 4 minutes to test time reduction for efficiency [5,15,16].

**Figure 1:**
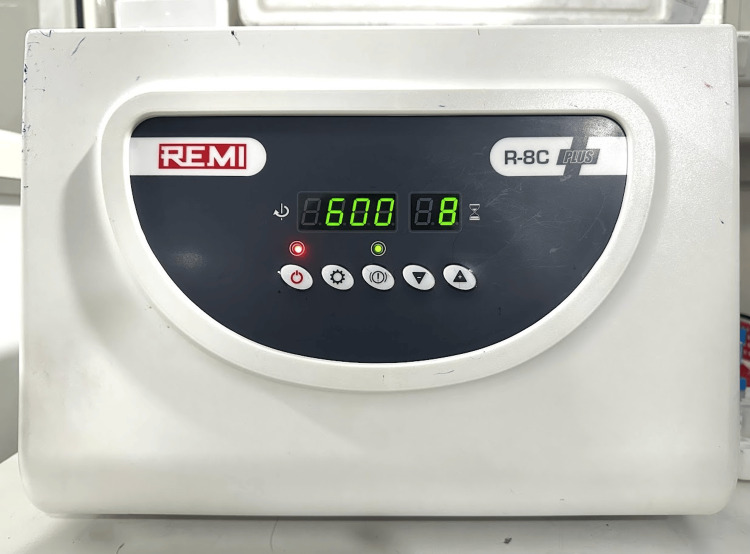
Remi R-8C Plus centrifuge set at RCF of 600 rpm for 8 minutes RCF: relative centrifugal force.

**Figure 2:**
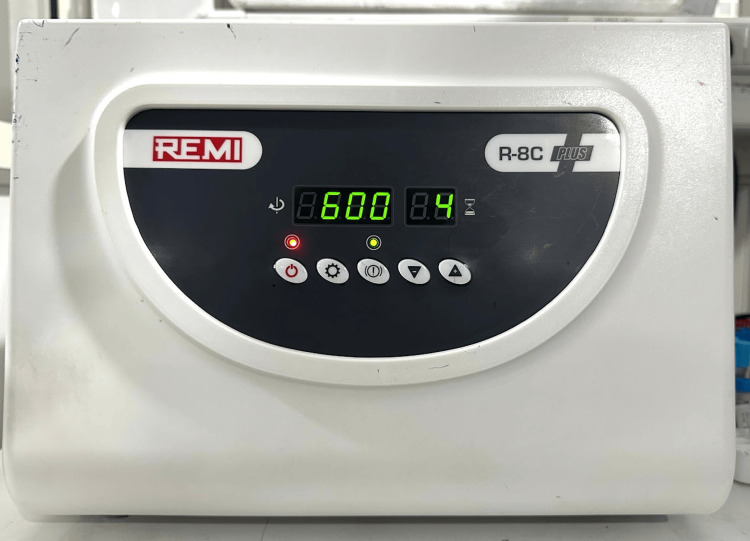
Remi R-8C Plus centrifuge set at RCF of 600 rpm for 4 minutes RCF: relative centrifugal force.

The study was conducted from July to December 2024 at the Department of Periodontics, Amrita School of Dentistry, Amrita Vishwa Vidyapeetham, Kochi, India, by utilizing standardized laboratory facilities, including a Remi R-8C Plus centrifuge, Sysmex XN-2000 Hematology Analyzer for flow cytometry, and DuoSet ELISA system for growth factor quantification. Figure 1 shows the Remi R-8C Plus centrifuge set at RCF of 600 rpm for 8 minutes, and Figure 2 shows the Remi R-8C Plus centrifuge set at RCF of 600 rpm for 4 minutes.

Peripheral blood was collected from 10 healthy male volunteers aged 20-30 years, recruited from the outpatient department. Only males were included to minimize hormonal influences in blood composition [17]. Written informed consent was obtained, and the study adhered to ethical guidelines approved by the institutional review board. The inclusion criteria were healthy males aged 20-30 years with no systemic conditions. Individuals with infectious diseases, excessive nicotine or alcohol use, anticoagulant therapy, current medications, or blood dyscrasias were excluded to ensure sample homogeneity [5,18].

**Figure 3:**
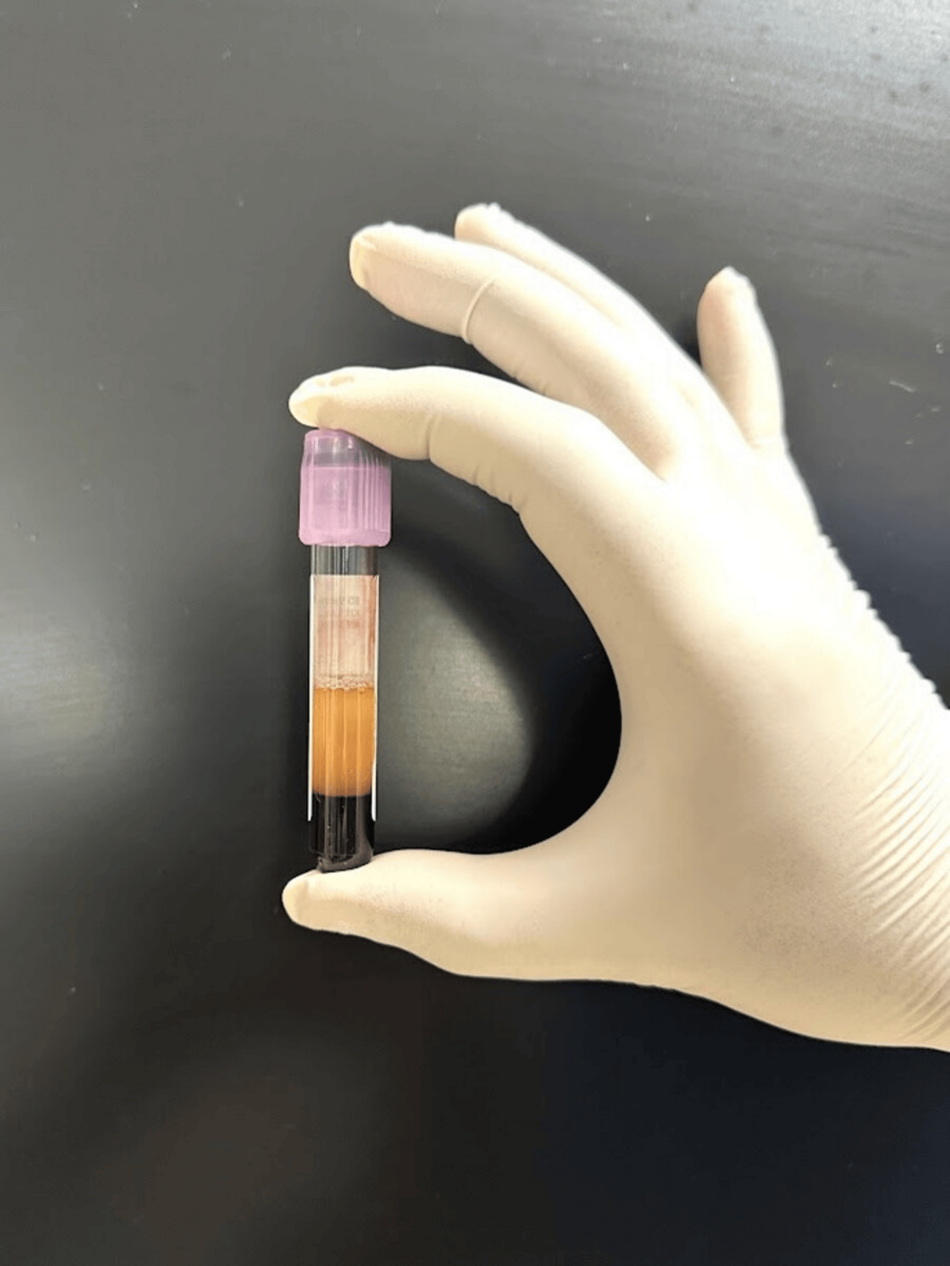
PRF obtained after 8 minutes PRF: platelet-rich fibrin.

**Figure 4:**
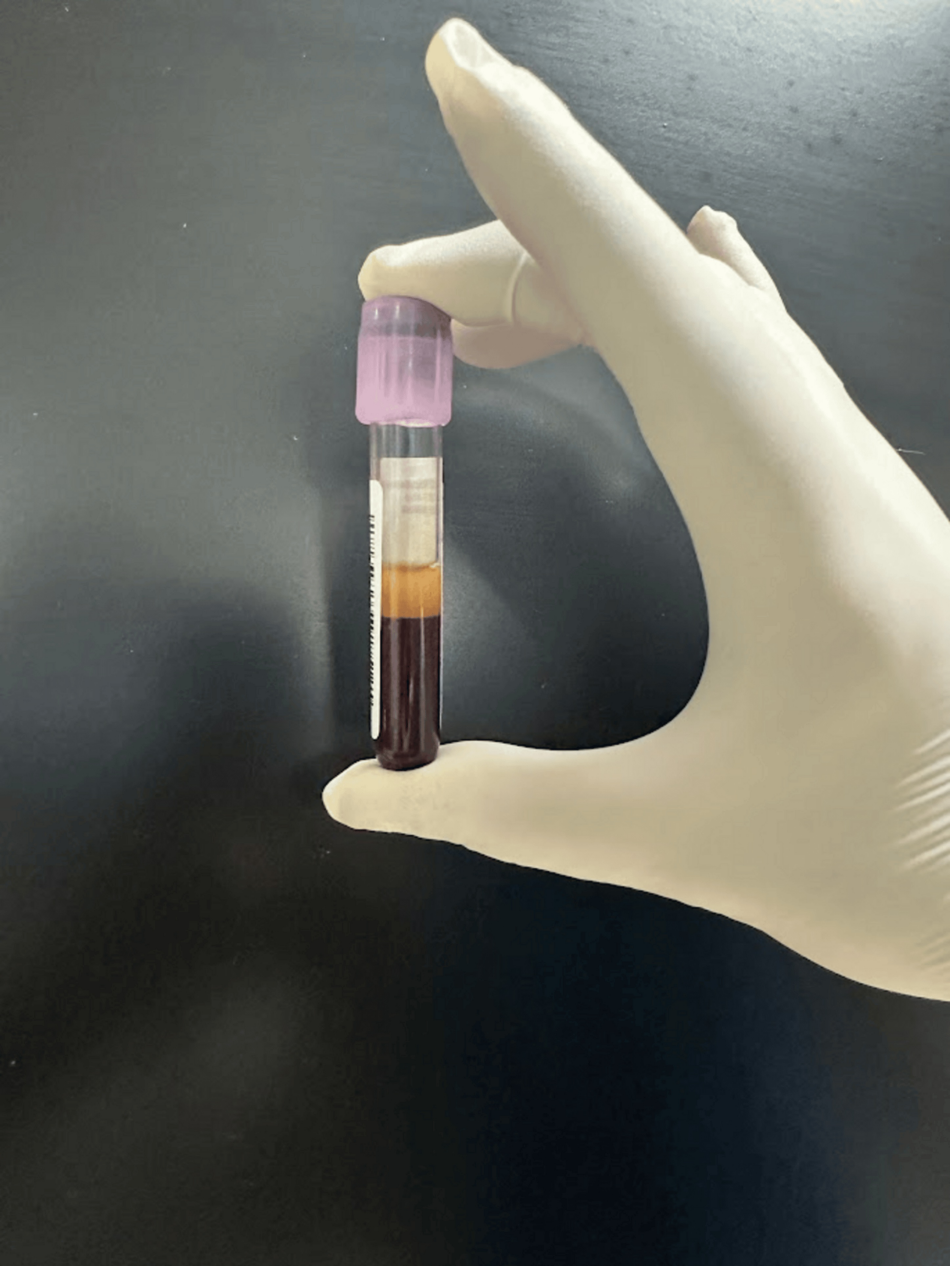
PRF obtained after 4 minutes PRF: platelet-rich fibrin.

Blood was collected in 15 mL Falcon tubes (Process for PRF, Nice, France) without anticoagulants. Centrifugation was performed using a Remi R-8C Plus with a fixed-angle rotor (radius 110 mm). Flow cytometry employed the Sysmex XN-2000, and growth factor analysis used the DuoSet ELISA system. Vacutainers and Dulbecco’s Modified Eagle Medium (Biochrom GmbH, Berlin, Germany) supported cellular and molecular analyses [19]. Ten milliliters of blood per sample were drawn into Falcon tubes and immediately centrifuged. Protocol I involved 8 minutes at 600 rpm (44 g), and Protocol II used 4 minutes at the same RCF. Processing occurred within 2-3 minutes of collection to prevent premature clotting, adhering to LSCC protocols. Centrifugation used standard acceleration and no brake [5]. Figure 3 and Figure 4 show PRF obtained after 8 and 4 minutes, respectively.

Four parameters were assessed: platelet count (cells/µL) via flow cytometry, leukocyte count (cells/µL) via flow cytometry, TGF-β concentration (ng/mL) via ELISA at 1 and 24 hours post-clotting, and VEGF concentration (pg/mL) via ELISA at 1 and 24 hours post-clotting. These parameters were selected for their roles in periodontal regeneration. Baseline whole blood leukocyte and platelet counts were verified to be within normal limits (leukocytes: 4-11 x 10^3^/µL; platelets: 150-450 x 10^3^/µL) prior to PRF processing [19,20].

**Figure 5:**
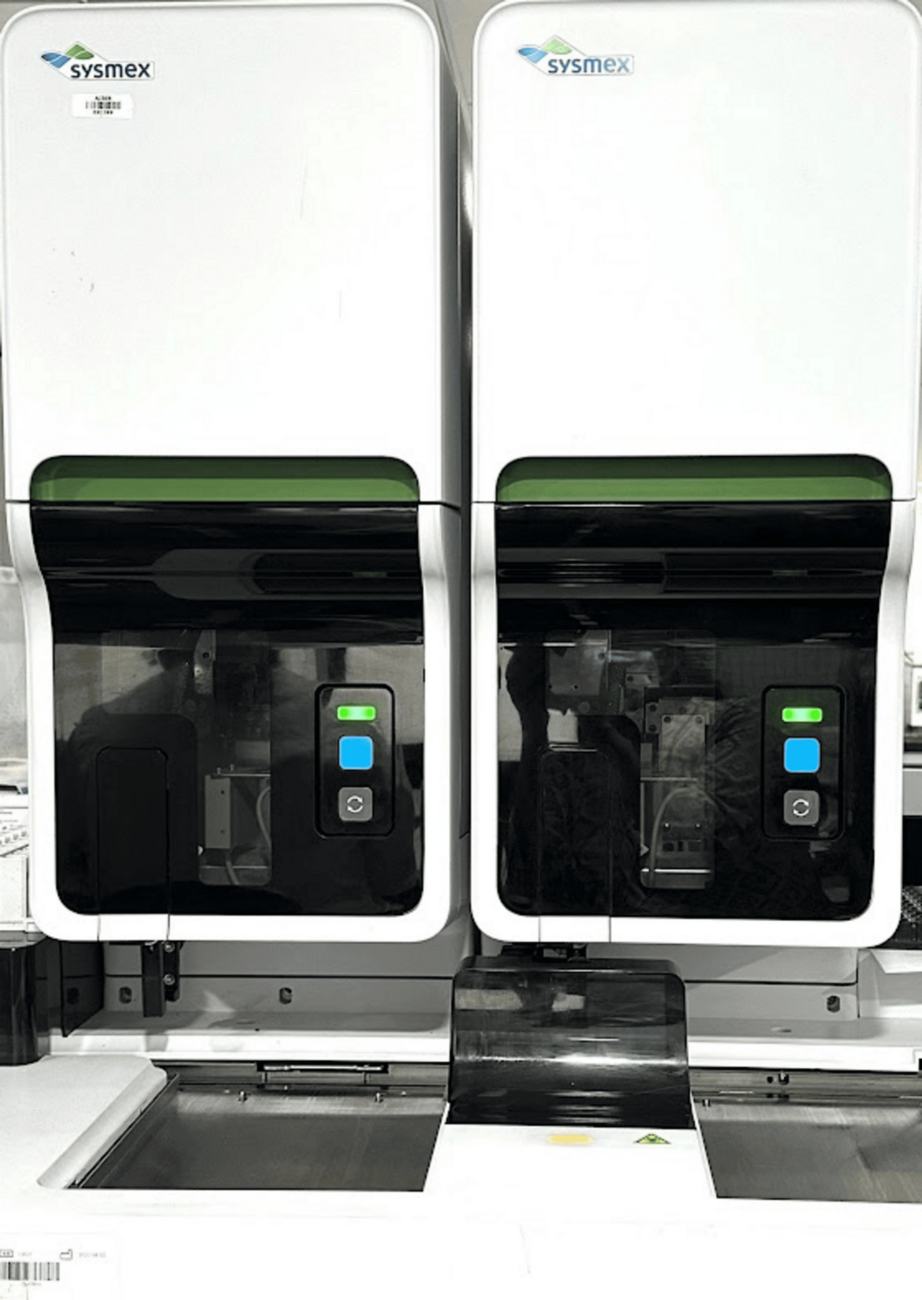
Sysmex XN-2000 used for flow cytometry

Fluid PRF samples were anticoagulated with EDTA and analyzed using the Sysmex XN-2000, employing fluorescence flow cytometry for precise leukocyte and platelet quantification [21]. The device is shown in Figure 5.

**Figure 6:**
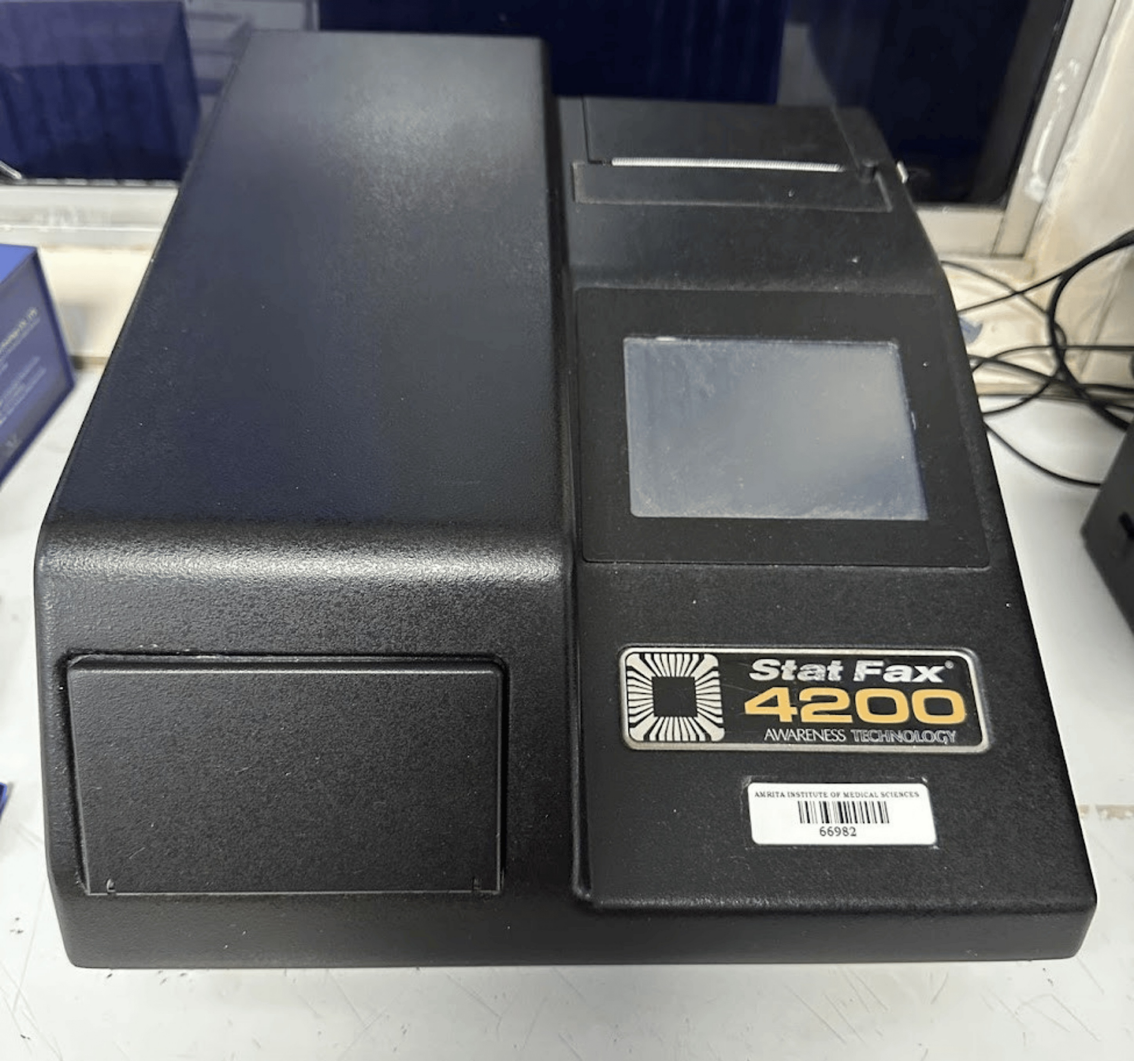
Stat Fax 4200 Microplate Reader for processing and analyzing ELISA

VEGF and TGF-β1 concentrations were measured using the DuoSet ELISA system. R&D Systems DuoSet kits were used (DY240-05 for TGF-β1, detection limit 31.2 pg/mL; DY293B-05 for VEGF, detection limit 15.6-31.2 pg/mL); assays were performed in triplicate. Fluid PRF was clotted at 37°C in cell culture plates with DMEM. Supernatants were collected at 1 and 24 hours post-clotting, and concentrations represent cumulative release (1h + 24h) to assess early dynamics. One hundred microliters of fluid PRF clotted with 900 µL DMEM at 37°C in 96-well plates; supernatants were collected at 1 and 24 hours [22]. A Stat Fax 4200 Microplate Reader (Figure 6) was used for processing and analyzing ELISA. 

**Figure 7:**
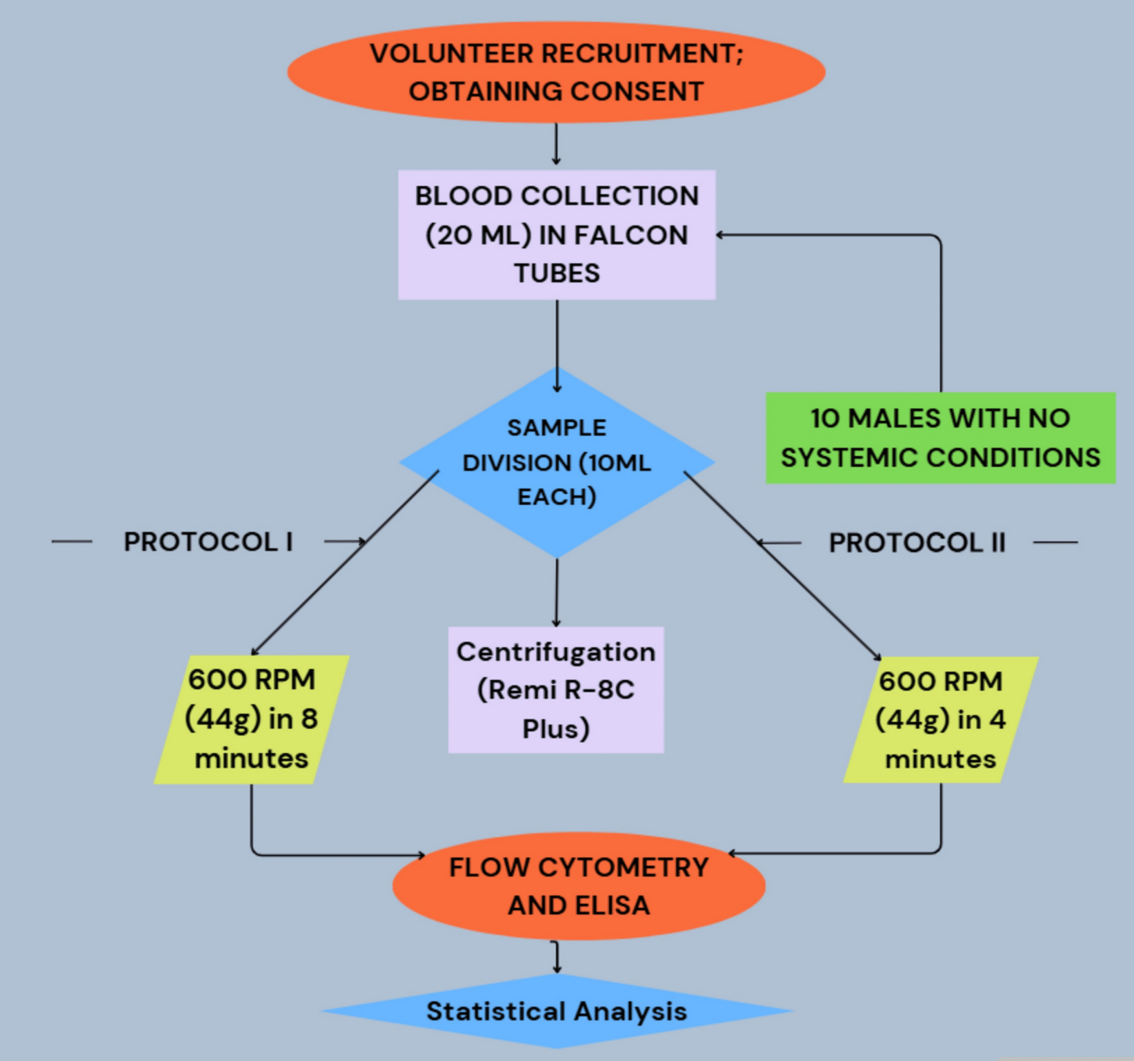
Flow diagram of the study methodology Image Credits: Dr. Shankar S Menon.

The methodology is detailed using a flow diagram as given in Figure 7.

Statistical analysis

The sample size was calculated a priori using GPower software (version 3.1.9.7; Heinrich-Heine-Universität Düsseldorf, Düsseldorf, Germany) for a Wilcoxon signed-rank test, targeting an effect size of 0.8 for platelet concentration (primary outcome), with alpha = 0.05 and power = 0.80, yielding n = 10. The minimum sample size was thus estimated at 10 participants. This aligns with resource constraints for an in vitro study. Data were analyzed using IBM SPSS Statistics for Windows (IBM Corp., Armonk, NY, USA). Non-normal data distribution was determined using the Shapiro-Wilk test, applied to each parameter (leukocytes, platelets, TGF-β, VEGF). This test was selected for its suitability with small sample sizes. The Wilcoxon signed-rank test compared paired observations between 8-minute and 4-minute protocols for leukocyte, platelet, TGF-β, and VEGF concentrations, with p ≤ 0.05 indicating significance. Non-parametric methods were chosen due to the small sample size (n = 10) and non-normal data distribution. Boxplots visualize data variability.

## Results

Ten blood samples were processed under Protocols I (8 minutes) and II (4 minutes), yielding 20 PRF samples. Leukocyte and platelet counts were quantified via flow cytometry, and TGF-β and VEGF concentrations via ELISA. Results were analyzed using the Wilcoxon signed-rank test, with boxplots illustrating data distribution. The results are presented in Table 1 and Table 2.

**Table 1:** Leukocyte and platelet counts (in ×10³ cells per microliter) for 10 platelet-rich fibrin (PRF) samples prepared using two different centrifugation times: 8 minutes and 4 minutes The data compares the effect of centrifugation duration on cell concentrations in PRF preparations when centrifuged at 600 rpm.

Sample no.	Platelet/leukocyte count (×10³ cells/µL)		
		8 minutes	4 minutes
1	Leukocyte	0.01	0.06
	Platelet	517	666
2	Leukocyte	0.04	0.1
	Platelet	530	742
3	Leukocyte	0.08	0.07
	Platelet	578	705
4	Leukocyte	0.04	0.07
	Platelet	480	541
5	Leukocyte	0.01	0.02
	Platelet	413	479
6	Leukocyte	0.04	0.9
	Platelet	646	745
7	Leukocyte	0.01	0.2
	Platelet	634	740
8	Leukocyte	0.05	0.07
	Platelet	595	691
9	Leukocyte	0.01	0.03
	Platelet	497	608
10	Leukocyte	0.07	0.21
	Platelet	813	901

**Table 2:** Concentrations of transforming growth factor-beta (TGF-β) and vascular endothelial growth factor (VEGF) in platelet-rich fibrin (PRF) samples prepared using two different centrifugation times: 8 minutes and 4 minutes The data compares the effect of centrifugation duration on the release of these growth factors in PRF preparations when centrifuged at 600 rpm.

Sample no.	Growth factor concentration		
		8 minutes	4 minutes
1	TGFB (ng/mL)	34.09	36.2
	VEGF (pg/mL)	2.1	6.1
2	TGFB (ng/mL)	37.78	23.46
	VEGF (pg/mL)	4.96	134.22
3	TGFB (ng/mL)	37.76	37.76
	VEGF (pg/mL)	7.43	10.86
4	TGFB (ng/mL)	35.16	36.43
	VEGF (pg/mL)	4.19	3.24
5	TGFB (ng/mL)	35.54	35.18
	VEGF (pg/mL)	5.15	3.43
6	TGFB (ng/mL)	38.51	37.38
	VEGF (pg/mL)	0.95	3.24
7	TGFB (ng/mL)	35.57	29.71
	VEGF (pg/mL)	5.34	12.77
8	TGFB (ng/mL)	21.22	17.63
	VEGF (pg/mL)	2.67	15.44
9	TGFB (ng/mL)	36.76	37.92
	VEGF (pg/mL)	4.19	7.05
10	TGFB (ng/mL)	37.4	38.64
	VEGF (pg/mL)	5.53	4.57

Leukocyte concentrations

**Figure 8:**
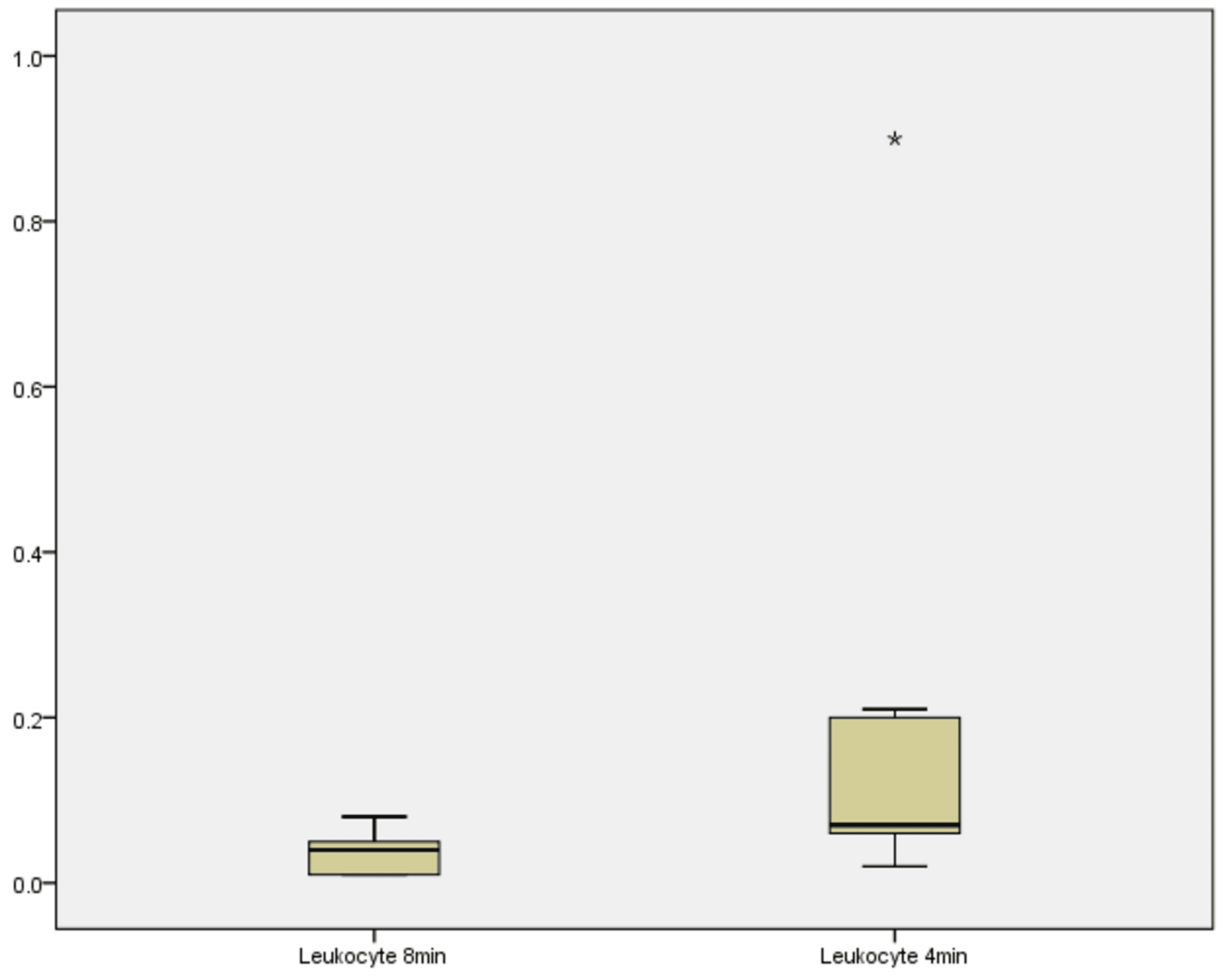
Boxplot showing leukocyte concentrations across 8 minutes and 4 minutes

Leukocyte concentrations were measured at both centrifugation durations across all 10 samples (Figure 8). At 8 minutes, values ranged from 0.01 to 0.08 × 10³ cells/µL, with a median of 0.04 × 10³ cells/µL. At 4 minutes, the range expanded to 0.02-0.9 × 10³ cells/µL, with a median of 0.07 × 10³ cells/µL. The Wilcoxon signed-rank test confirmed a statistically significant increase in leukocyte concentration at 4 minutes (Z = -2.654, p = 0.008). Nine samples exhibited higher leukocyte counts at the reduced time, with only one showing a decrease and no ties observed. This robust increase suggests that shortening centrifugation time enhances leukocyte retention in PRF, likely due to reduced cellular sedimentation at lower durations [4,5].

Platelet concentrations

**Figure 9:**
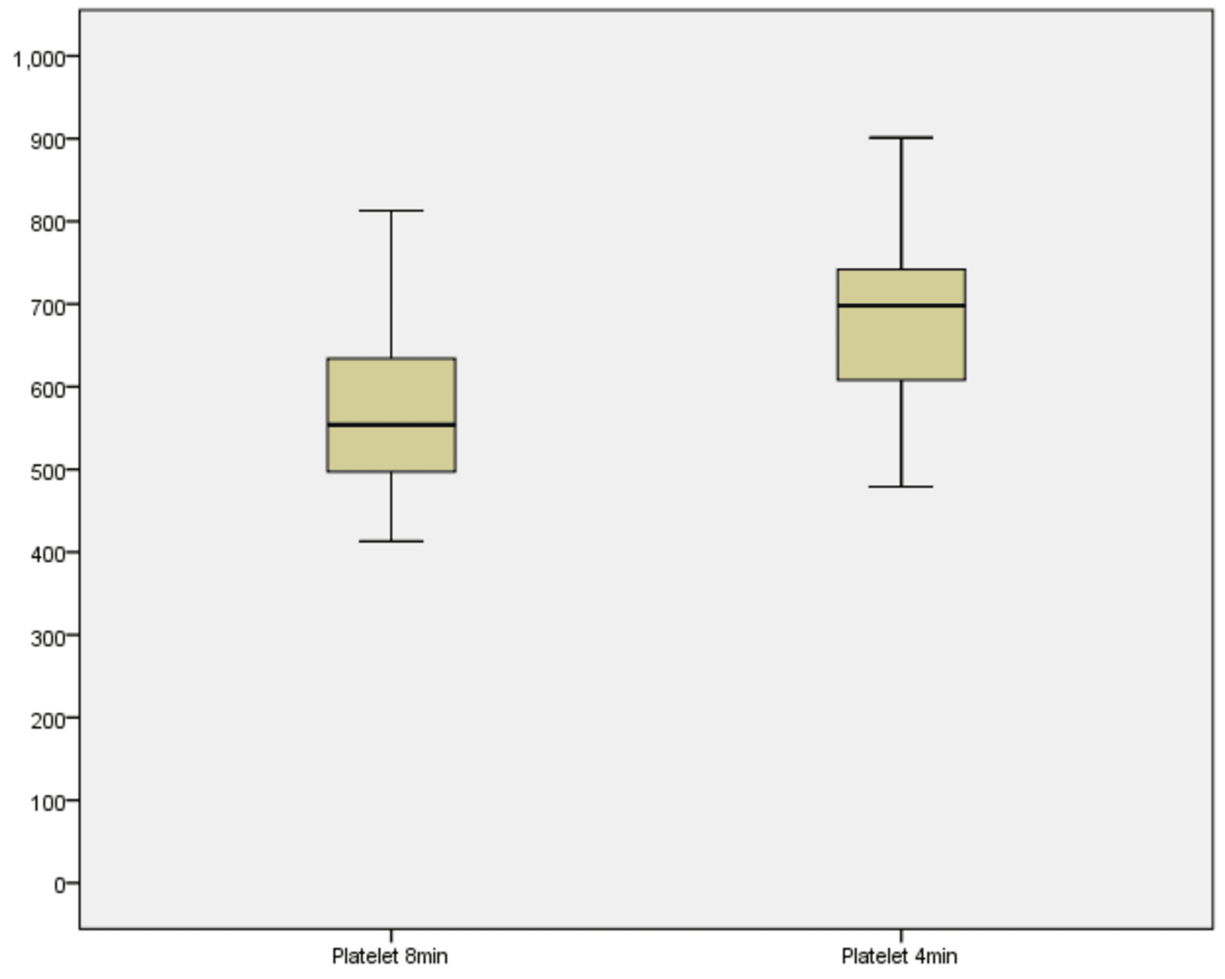
Boxplot showing platelet concentrations across 8 minutes and 4 minutes

Platelet counts were similarly assessed at both time points (Figure 9). At 8 minutes, concentrations ranged from 413 to 813 × 10³ cells/µL, with a median of 578 × 10³ cells/µL. At 4 minutes, the range increased to 479-901 × 10³ cells/µL, with a median of 691 × 10³ cells/µL. The Wilcoxon signed-rank test indicated a significant rise in platelet concentration at 4 minutes (Z = -2.803, p = 0.005). All 10 samples demonstrated higher platelet counts at the shorter duration, with no decreases or ties noted. This consistent elevation implies that reducing centrifugation time optimizes platelet yield, potentially by minimizing excessive compaction and loss of platelets during fibrin matrix formation [7,8].

TGF-β concentrations

**Figure 10:**
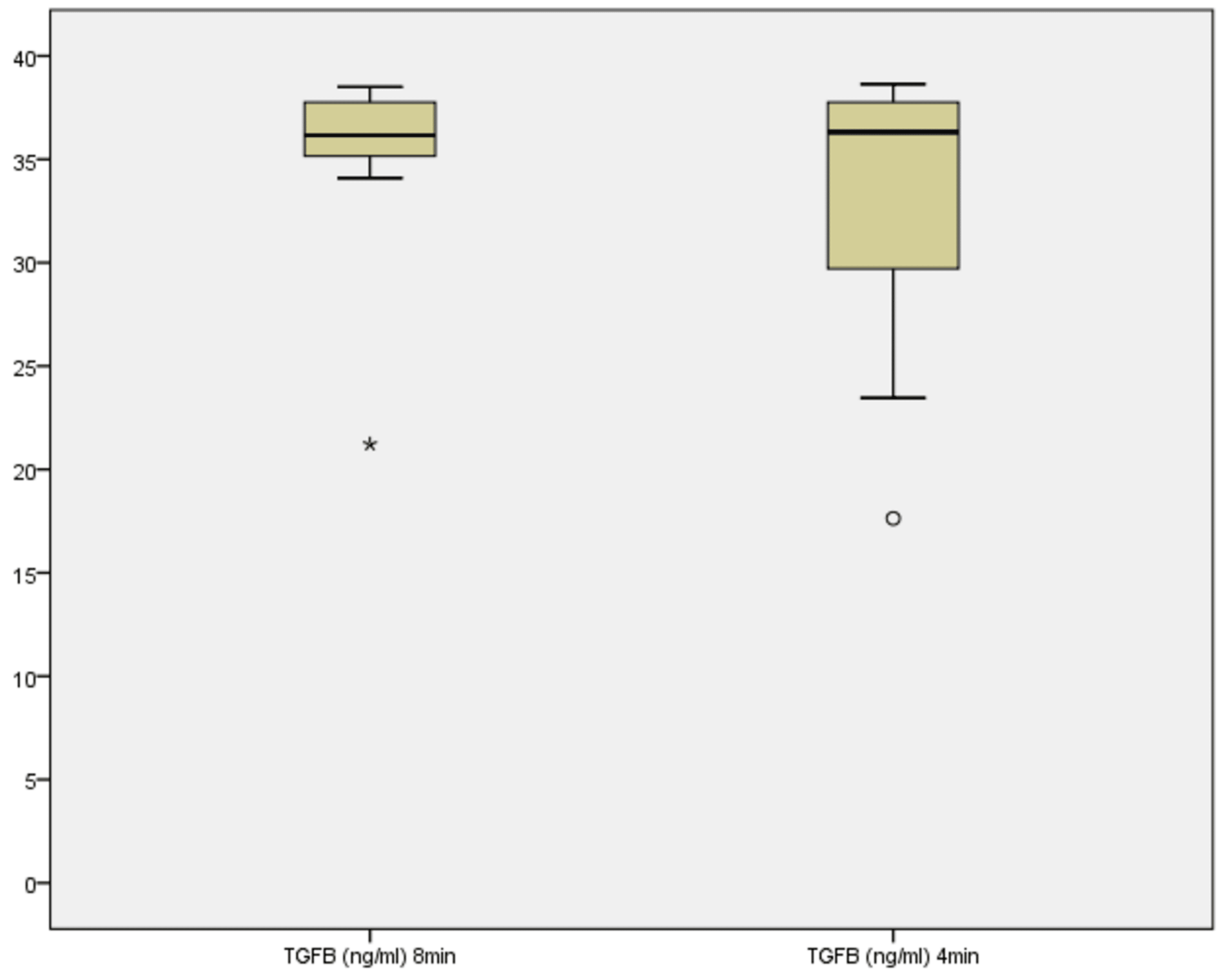
Boxplot showing TGF-β concentrations across 8 minutes and 4 minutes TGF-β: transforming growth factor-beta.

TGF-β concentrations were measured in nanograms per milliliter (ng/mL) across all 10 samples (Figure 10). At 8 minutes, levels ranged from 21.22 to 38.51 ng/mL, with a median of 35.57 ng/mL. At 4 minutes, the range was 17.63-38.64 ng/mL, with a median of 36.43 ng/mL. Although the Wilcoxon signed-rank test yielded a non-significant p-value (Z = -0.533, p = 0.594), a closer examination revealed that four samples showed an increase at 4 minutes, five showing a decrease, and one remained unchanged. The median increase from 35.57 to 36.43 ng/mL suggests a subtle upward trend in TGF-β concentration. This may indicate that shorter centrifugation enhances the release or preservation of TGF-β in some samples, possibly due to less structural disruption of the fibrin matrix [8,20].

VEGF concentrations

**Figure 11:**
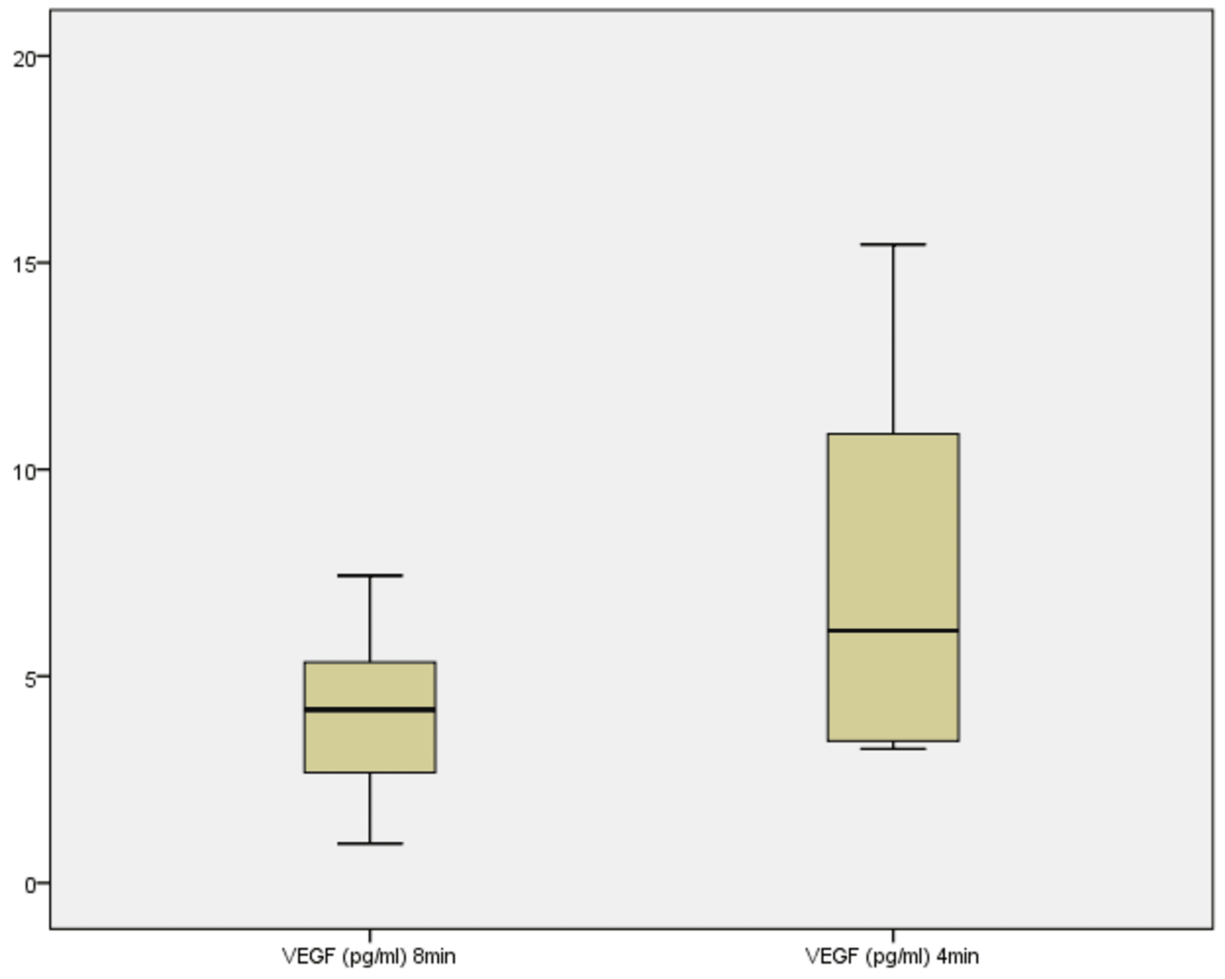
Boxplot showing VEGF concentrations across 8 minutes and 4 minutes VEGF: vascular endothelial growth factor.

VEGF concentrations were initially evaluated across all 10 samples in picograms per milliliter (pg/mL) (Figure 11). At 8 minutes, levels ranged from 0.95 to 7.43 pg/mL, with a median of 4.19 pg/mL. At 4 minutes, the range widened significantly to 3.24-134.22 pg/mL, with a median of 6.10 pg/mL. The Wilcoxon signed-rank test showed a borderline significant increase (Z = -1.955, p = 0.051). Seven samples exhibited higher VEGF levels at 4 minutes, while three showed a decrease. The substantial increase in median value and the extended upper range suggest that reducing centrifugation time markedly enhances VEGF concentration, possibly due to greater retention of growth factor-rich plasma components.

**Figure 12:**
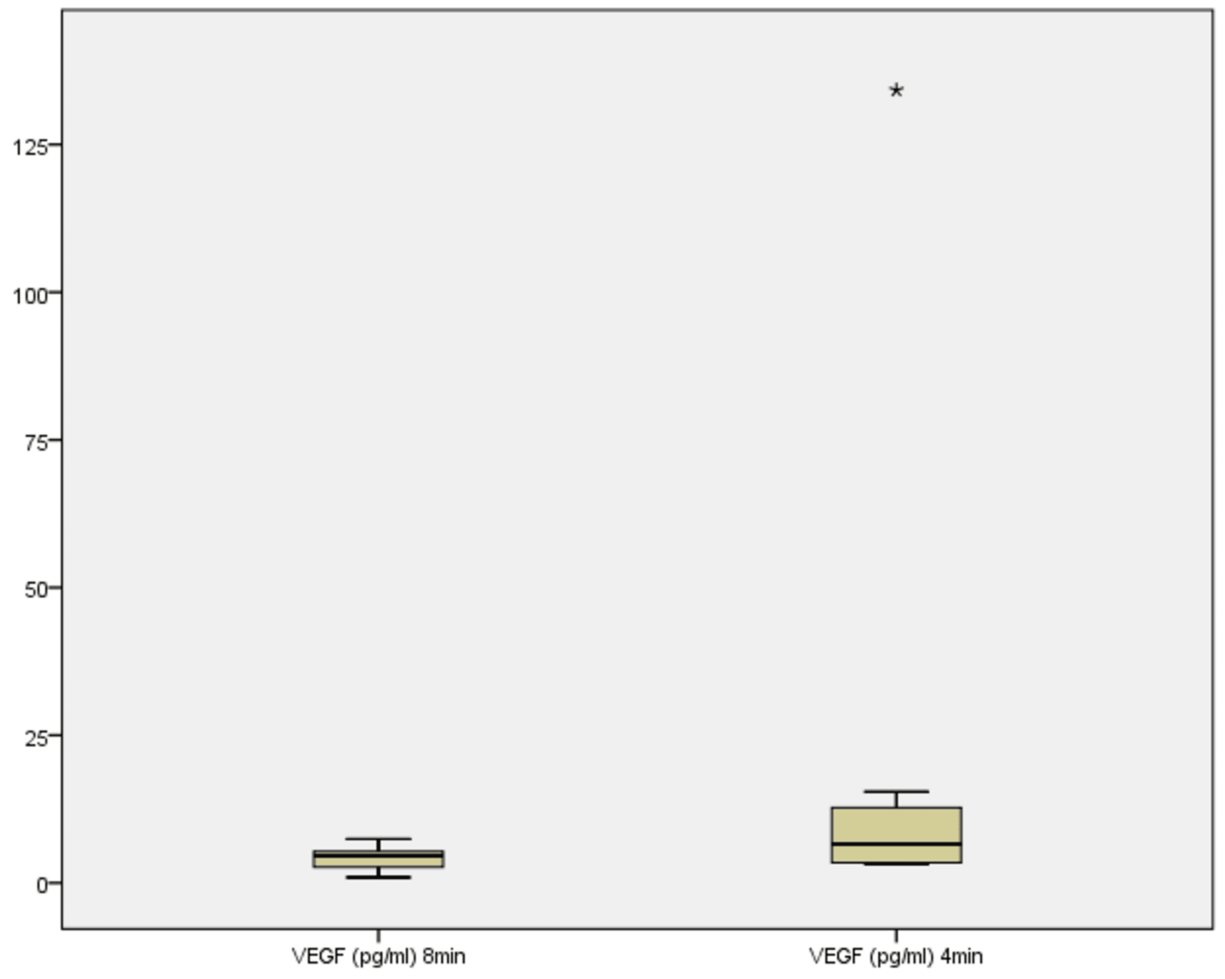
Boxplot showing VEGF concentrations across 8 minutes and 4 minutes in nine samples (one sample excluded) VEGF: vascular endothelial growth factor.

One extreme VEGF value in one sample was both retained and excluded, and separate statistical analyses were performed, with and without this outlier sample. Excluding the outlier (134.22 pg/mL), VEGF concentrations were re-analyzed for nine samples (Figure 12). At 8 minutes, the range was 0.95-7.43 pg/mL, with a median of 4.19 pg/mL. At 4 minutes, the range adjusted to 3.24-15.44 pg/mL, with a median of 6.10 pg/mL. The Wilcoxon signed-rank test again indicated a near-significant increase (Z = -1.955, p = 0.051), with six samples showing higher values at 4 minutes and three showing decrease. The consistent median increase and the majority of samples trending upward reinforce the interpretation that shorter centrifugation time augments VEGF concentration, reflecting an enhanced release mechanism at 4 minutes [11,13,18]. Figure 12 illustrates the Boxplot showing VEGF concentrations across 8 min and 4 min in 9 samples (1 sample excluded).

The results collectively indicate that reducing centrifugation time from 8 minutes to 4 minutes at 600 rpm significantly increases leukocyte (p = 0.008) and platelet (p = 0.005) concentrations in PRF. While TGF-β showed no statistically significant change (p = 0.594), the median increase and upward trend in several samples suggest a potential enhancement in growth factor availability. Similarly, VEGF exhibited a near-significant increase (p = 0.051) in both 10-sample and 9-sample analyses, with a clear majority of samples showing higher concentrations at 4 minutes. These findings postulate that shorter centrifugation time at low-speed RCF consistently enhances all four parameters (leukocytes, platelets, TGF-β, and VEGF), likely by reducing cellular and molecular sedimentation, preserving fibrin matrix integrity, and enhancing the release of growth factors. This optimization could enhance PRF’s regenerative potential in periodontal applications.

## Discussion

This study demonstrates that reducing centrifugation time from 8 to 4 minutes at 600 rpm (44 g) significantly enhances PRF’s regenerative composition, with increased leukocyte (p = 0.008) and platelet (p = 0.005) concentrations, and a near-significant rise in VEGF (p = 0.051), accompanied by a modest upward trend in TGF-β (p = 0.594). These findings align with the LSCC, which emphasizes reducing RCF to optimize cell and growth factor yields [9]. By focusing on time reduction rather than solely on force, this study advances PRF optimization and highlights its clinical potential in periodontal regeneration, including applications in socket preservation, intrabony defect repair, and gingival recession management.

The significant increase in leukocyte concentration after 4 minutes supports previous findings by Ghanaati et al., who demonstrated that lower centrifugal forces preserve leukocytes crucial for immunomodulation and tissue remodeling [4]. Leukocytes, especially neutrophils, regulate early inflammation and orchestrate tissue-healing environments. Hauser et al. reported similar improvements in socket preservation outcomes when leukocyte-rich PRF was used, with histologic evidence of reduced alveolar bone loss [22]. This increase likely results from reduced sedimentation during shorter spins, preventing leukocyte entrapment at lower layers, which is a limitation of extended centrifugation noted by Anitua et al. [6].

Similarly, the robust increase in platelet concentration corroborates Kobayashi et al.’s observations that optimized PRF protocols improve platelet entrapment and preserve platelet-derived growth factors such as PDGF and TGF-β [7]. Platelets play a vital role in matrix formation, fibroblast proliferation, and angiogenesis, all of which are critical for periodontal regeneration. Sharma and Pradeep reported substantial clinical attachment gain and defect fill when PRF was used in mandibular furcation defects, reflecting platelet-driven regeneration [10]. The current findings, therefore, confirm that reduced centrifugation time enhances platelet retention by minimizing fibrin over-compaction and shear stress [8].

The near-significant increase in VEGF parallels Miron et al.’s injectable PRF (i-PRF) studies, where reduced centrifugation improved VEGF concentration and angiogenic potential [19]. VEGF promotes revascularization, essential for nutrient delivery and soft-tissue viability. Panda et al. demonstrated that PRF-enhanced angiogenesis significantly improved gingival recession coverage and healing response [23]. The broader VEGF range observed in the present study, even after excluding an outlier, reinforces the relationship between centrifugation parameters and growth factor enrichment, as it suggests enhanced growth factor liberation, possibly due to reduced cellular stress. This augmentation could accelerate angiogenesis in periodontal applications [13,18].

Although TGF-β changes were not statistically significant, a subtle median increase suggests improved preservation of this growth factor. Lundquist et al. reported that shorter centrifugation enhances fibrin density and stabilizes embedded growth factors [8]. TGF-β plays a central role in extracellular matrix synthesis, collagen formation, and fibroblast proliferation, which are key elements in periodontal defect repair. These results suggest that while growth factor kinetics vary, the overall biological potency of PRF is augmented under shorter centrifugation durations [11,20]. Future studies with larger cohorts could clarify TGF-β’s response to time reduction.

The clinical relevance of optimizing fluid PRF protocols, such as the reduced centrifugation time evaluated in this study, is further supported by recent evidence on injectable PRF (i-PRF) applications in periodontal soft-tissue management. A systematic review and meta-analysis of randomized controlled trials demonstrated that i-PRF injections significantly increase gingival thickness (mean difference 0.12-0.38 mm) in patients with thin gingival phenotype, with greater keratinized tissue width gains observed with four-session protocols compared to three sessions; combining i-PRF with microneedling further enhanced gingival thickness outcomes, highlighting the importance of protocol standardization for maximal regenerative effects [24]. Similarly, a split-mouth randomized clinical trial comparing injectable PRF to hyaluronic acid for thin gingival phenotype management reported favorable improvements in gingival parameters with i-PRF, underscoring its autologous advantages in promoting tissue augmentation without foreign materials [25]. Although one study evaluating platelet-rich plasma (rather than PRF) injections during orthodontic tooth movement suggested potential acceleration of retraction rates [26], the distinct preparation and composition of PRF variants like those optimized here may offer superior biocompatibility and sustained growth factor release for periodontal regeneration. Collectively, these findings reinforce the translational potential of low-speed, time-optimized PRF protocols in enhancing clinical outcomes for gingival phenotype modification and related therapies.

The practical advantage of a 4-minute spin lies in its efficiency. Shorter centrifugation halves chair-side preparation time, aligning with Miron et al.’s emphasis on clinically practical protocols that balance yield and convenience [19]. The procedure enables better synchronization with surgical workflows and reduces handling time without compromising biological quality. The protocol’s balance of cellular and molecular enrichment with operational streamlining, thus, positions it as a refined approach for periodontal therapy.

Study limitations

This ex vivo design limits extrapolation to in vivo periodontal environments where cellular interactions and vascular dynamics influence regenerative outcomes [14]. The small sample size (n = 10) restricts statistical power and generalizability. It may not fully capture inter-individual variability influenced by subclinical conditions such as recent physical activity or nutritional status, despite strict exclusion criteria. Inclusion of only male participants may overlook gender-based physiological variations in platelet or leukocyte content [17]. Moreover, assay validation was limited to manufacturer specifications; independent validation was not performed. Only one centrifuge model and rotor configuration (Remi R-8C Plus, fixed-angle rotor, radius 11 cm) was used, which may limit generalizability to other centrifuge systems. Total leukocyte count was assessed without differential typing, limiting insights into specific subsets like monocytes/macrophages, which may play a more direct role in regeneration [11]. Additionally, the study analyzed growth factor release at only 1 and 24 hours; long-term kinetics are crucial to evaluate sustained release and bioactivity [16].

Future scope

Future investigations should involve larger, gender-balanced cohorts and varied RCF values across broader age ranges (e.g., 40-60 years) to reflect typical periodontal patients to validate these findings [17]. Quantification of other regenerative mediators, such as platelet-derived growth factor (PDGF) and bone morphogenetic protein-2 (BMP-2), may further elucidate PRF’s biological potential [18]. Clinical trials assessing the 4-minute protocol’s outcomes in periodontal defect regeneration, ridge preservation, and mucogingival procedures would substantiate its translational relevance. Future studies must also include larger, gender-balanced cohorts.

## Conclusions

Reducing centrifugation time from 8 to 4 minutes at 600 rpm (44 g) significantly improves PRF’s regenerative composition by increasing leukocyte and platelet concentrations and enhancing VEGF levels, with a modest rise in TGF-β. These improvements result from reduced sedimentation and improved fibrin-network integrity, enabling higher cellular and growth factor yields. The 4-minute protocol, though a preliminary biochemical trend, demonstrates a practical advantage for clinicians, offering both time efficiency and biological enhancement, making it suitable for various periodontal regenerative applications such as socket preservation, intrabony defect repair, and soft-tissue grafting. Although limited by its ex vivo nature and small sample size, this study provides a sound basis for optimizing centrifugation parameters in PRF preparation. Further clinical and biochemical studies are needed to confirm these benefits and expand the LSCC into routine periodontal practice.
